# Crystallization and Thermal Behaviors of Poly(ethylene terephthalate)/Bisphenols Complexes through Melt Post-Polycondensation

**DOI:** 10.3390/polym12123053

**Published:** 2020-12-19

**Authors:** Shichang Chen, Shangdong Xie, Shanshan Guang, Jianna Bao, Xianming Zhang, Wenxing Chen

**Affiliations:** 1 Key Laboratory of Advanced Textile Materials and Manufacturing Technology, Ministry of Education, Zhejiang Sci-Tech University, Hangzhou 310018, China; scchen@zstu.edu.cn; 2 Zhejiang Provincial Key Laboratory of Fiber Materials and Manufacturing Technology, Zhejiang Sci-Tech University, Hangzhou 310018, China; 3 School of Materials Science and Engineering, Zhejiang Sci-Tech University, Hangzhou 310018, China; xshangdong96@163.com (S.X.); gssysb@163.com (S.G.); wxchen@zstu.edu.cn (W.C.)

**Keywords:** poly(ethylene terephthalate), bisphenol, crystallization kinetics, thermal property, melt polycondensation

## Abstract

Three kinds of modified poly(ethylene terephthalate) (PET) were prepared by solution blending combined with melt post-polycondensation, using 4,4′-thiodiphenol (TDP), 4,4′-oxydiphenol (ODP) and hydroquinone (HQ) as the bisphenols, respectively. The effects of TDP, ODP and HQ on melt post-polycondensation process and crystallization kinetics, melting behaviors, crystallinity and thermal stability of PET/bisphenols complexes were investigated in detail. Excellent chain growth of PET could be achieved by addition of 1 wt% bisphenols, but intrinsic viscosity of modified PET decreased with further bisphenols content. Intermolecular hydrogen bonding between carbonyl groups of PET and hydroxyl groups of bisphenols were verified by Fourier transform infrared spectroscopy. Compare to pure PET, both the crystallization rate and melting temperatures of PET/bisphenols complexes were reduced obviously, suggesting an impeded crystallization and reduced lamellar thickness. Moreover, the structural difference between TDP, ODP and HQ played an important role on crystallization kinetics. It was proposed that the crystallization rate of TDP modified PET was reduced significantly due to the larger amount of rigid benzene ring and larger polarity than that of PET with ODP or HQ. X-ray diffraction results showed that the crystalline structure of PET did not change from the incorporation of bisphenols, but crystallinity of PET decreased with increasing bisphenols content. Thermal stability of modified PET declined slightly, which was hardly affected by the molecular structure of bisphenols.

## 1. Introduction

Poly(ethylene terephthalate) (PET) is one of the most widely used linear semicrystalline thermoplastic polyester in many industrial and everyday applications because of its good mechanical and thermal properties, non-toxicity, processing low energy requirements and high chemical resistance [1,2,3]. Therefore, PET has been extensively used for the manufacture of beverage bottles, packing films, engineering plastics, industrial fibers and so on [4]. As a semicrystalline polymer, the crystallization and its degree of crystallinity play an important role on applications, which would highly affect physical and mechanical properties [5,6,7,8,9,10,11,12]. Generally speaking, the introduction of other polymers, nanofillers, comonomers and reactive functional groups are commonly used technique to modify the crystallization behaviors by means of copolymerization, blending, etc.

Tremendous effort has been ongoing to develop PET production methods by incorporation other polymers and adjusted crystallization kinetics of PET were characterized. It was found that in PET/PLA blends, the degree of crystallinity of PET was reduced by blending with polylactic acid (PLA) [13]. A more pronounced effect was observed in the crystallization rate of PET by the presence of PLA. It was proposed that PLA chains could act as barriers against movements of PET chains in diffusing through the surface of growing crystals [14]. In contrast, as in the polypropylene (PP)/PET blends, both of the crystallinity of PP and PET was enhanced [15]. With the addition of polyamide 56 (PA56) to PET, crystallization rate of PET was increased and increment was due to a nucleating effect by the early formed PA56 crystals [16]. Besides, both PA56 and PET exhibited smaller crystals compared large crystals with pure polymers. Several efforts have been made to regulate the crystallization rate of PET by inclusion of nanofillers [17,18]. Some authors found that SiO_2_ nanoparticles [19,20], modified graphene oxide [21], multiwalled carbon nanotubes [22,23], polyhedral oligomeric silsesquioxanes [24,25] and calcium carbonate [26] could act as a heterogeneous nucleating agent and increase the crystallinity of PET.

PET is produced from petroleum-based monomers through a chemical process involving condensation reactions. In this sense, except for physical blending, PET can be copolymerized with various polymers and exhibit highly sensitive crystallization kinetic toward inclusion of other foreign monomers [27,28,29]. Related studies have revealed that addition of low content of other kinds of diols, substituted to the main raw diols of PET, could reduce crystallinity and crystallization rate. For example, it has been reported that incorporating low content of 1,3-propanediol, 2-methyl-1,3-propanediol and 2,2-dimethyl-1,3-propanediol into PET backbone could reduce the crystallinity due to the decreased macromolecular regularity of PET [30,31,32]. Zhou et al. added sodium-5-sulfo-bis-(hydroxyethyl)-isophthalate and 2,2-dimethyl-1,3-propanediol to improve the hydrophilicity and dyeability of PET [33]. Zhao et al. synthesized PET-based ionomers with flame retardant and antidripping by melt polycondensation using a phosphorus-containing ionic monomer as an end-capping agent into the PET chain end [34].

Bisphenol, endowing the same dihydroxyl-terminal structure as diols, can serve as a substitution of diol to react with diacid. As a result, it was often used to mediate the crystallization behaviors and physical properties of polyesters, such as polybutylene terephthalate (PBT) [35], polybutylene succinate (PBS) [36], polycaprolactone (PCL) [37] and so on. In previous study, the overall isothermal crystallization rate and growth rate of spherulites of PBS were depressed by bisphenol A [36]. In the case of PBT, the crystallinity and crystal size were reduced in the presence of TDP [35]. Besides, the thermal and mechanical properties of poly (L-lactic acid) PLLA and poly(3-hydroxybutyrate) [P(3HB)] were greatly influenced through blending with 4,4′-thiodiphenol (TDP) [38,39]. Despite the extensive literature published regarding the crystallization behavior of PET containing systems, very few studies have reported crystallization behaviors of PET/bisphenols complexes. In this study, several selected bisphenols with different molecular structures were incorporated into PET by solution blending combined with melt post-polycondensation. The main aim of this work is to evaluate the effect of a small amount of bisphenols on the intrinsic viscosity, crystallization kinetics, melting behavior, thermal property and crystallinity of PET. A variety of characterization methods were used including differential scanning calorimetry (DSC), thermogravimetric analysis (TGA) and two-dimensional X-ray diffraction (2D XRD). Furthermore, roles of the bisphenol structure and cooling rate of the nonisothermal crystallization process on the crystallization kinetics were investigated in detail. Using modified Avrami equation, the nonisothermal crystallization kinetic of the bisphenols-modified PET systems was explored.

## 2. Materials and Methods

### 2.1. Materials

**Scheme 1:**
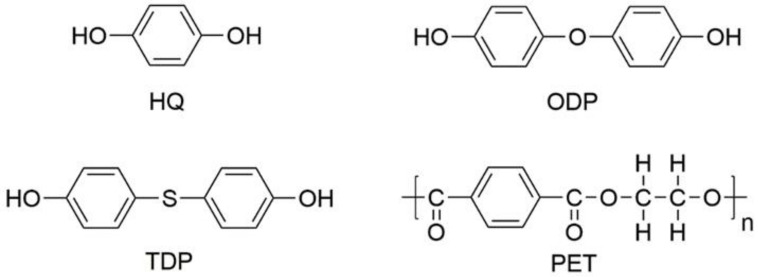
The chemical structure of bisphenols and PET.

PET chips with an intrinsic viscosity of 0.68 dL/g was kindly provided by Zhejiang Guxiandao Green Fiber Co., Ltd. (Hangzhou, China), TDP (AR grade), ODP (AR grade), HQ (AR grade) and 1,1,1,3,3,3-hexafluoroisopropyl alcohol (HFIP, LC grade) were purchased from Fluorochem (Derby, UK), molecular structures of four bisphenols were shown as Scheme 1. Phenol (AR grade) and 1,1,2,2-tetrachloroethane (AR grade) used as mixed solvent were bought from Shanghai Aladdin Bio-Chem Technology Co., Ltd. (Shanghai, China)

### 2.2. Sample Preparation

In order to make the bisphenols and PET fully and evenly mixed, the solution blending method was utilized. Prior to solution blending, both PET chips and bisphenols were dried in a vacuum at 90 °C for 24 h to remove as much residual moisture as possible. Bisphenols were weighed accurately and dissolved into HFIP solutions of PET chips to obtain samples. Four weight concentrations of bisphenols in PET were selected: 0, 1, 2 and 4% wt/wt. To reach a uniform dispersion in polymer matrix, the solutions were stirred evenly and slowly to evaporate the solvent. Subsequently, the resulting mixtures were dried in a vacuum at 120 °C for 24 h. The blends were hot-pressed into the designed film thickness (1 mm) sheet using a press vulcanizer (S(X)LB-350 × 350-25) (Suyan, Changzhou, China). The melt post-polycondensation experiments were carried out in a glass drier at 270 °C and 200 Pa for 60 min. At the end of reaction, the materials were taken out and placed in water to let it cool down.

### 2.3. Characterization

Viscosified PET sample was dissolved into phenol-tetrachloroethane (50/50 wt/wt) solutions. After dissolution, the intrinsic viscosity (IV) of pure PET and PET/bisphenols complexes was measured with a VISCO 070 Ubbelohde viscometer (Julabo, Seelbach, Germany) at 25 °C and averaged by three times.

Fourier transform infrared (FTIR) analysis was carried out on a Nicolet 5700 FTIR spectrometer (Thermo Electron, Waltham, Massachusetts, USA, resolution: 0.09 cm^−1^) to detect the functional groups of PET/TDP. The sample was prepared by KBr tablet and the spectra were recorded at room temperature.

Crystallization and melting behaviors of all samples were studied by means of a differential scanning calorimeter (DSC; Mettler Toledo, Zurich, Switzerland). Dry nitrogen was used as a purge gas at a rate of 45 mL/min during all measurements. For non-isothermal crystallization, samples of 6–8 mg encapsulated in aluminum pans were first heated from 25 to 280 °C and held for 5 min to eliminate any previous thermal history. Subsequently, the samples were cooled to 25 °C at different cooling rates of 5, 10, 20 and 30 °C/min, followed by a second heating to 280 °C at a constant cooling rate of 10 °C/min to evaluate their melting behaviors. Thermal stability analysis of all samples was performed on a thermal gravimetric analyzer (TGA, Mettler Toledo, Zurich, Switzerland), under nitrogen as DSC measurements. Samples of 7–8 mg were heated from 25 to 600 °C at a heating rate of 10 °C/min.

The crystalline structure and crystallinity of all specimens were analyzed by using two-dimensional wide-angle X-ray diffractions (WAXDs) with a D8 Discover diffractometer (Bruker, Karlsruhe, Germany). Before testing, samples were placed in a vacuum oven at 200 °C for 12 h crystallization. The wavelength of X-ray was 0.154 nm. The measurements were operated at a voltage of 40 kV and current of 40 mA. Coupled 2*θ*/*θ* was selected as a scanning mode and the test was divided into three steps (2*θ* = 20, 40, 60°). The scan time per step was 70 s and total time was 210 s. The diffraction patterns were fitted using a deconvolution procedure to fit all crystalline peaks and amorphous background. On the basis of the WAXD data, the crystallinity was estimated by comparing the diffraction area of amorphous peak at total areas of crystalline peaks, i.e., *X*_c_ = ∑*A*_c_/(∑*A*_c_ + ∑*A*_a_) and the parameters for each crystalline peak (width) could be also obtained.

## 3. Results and Discussion

### 3.1. Melt Post-Polycondensation

**Figure 1:**
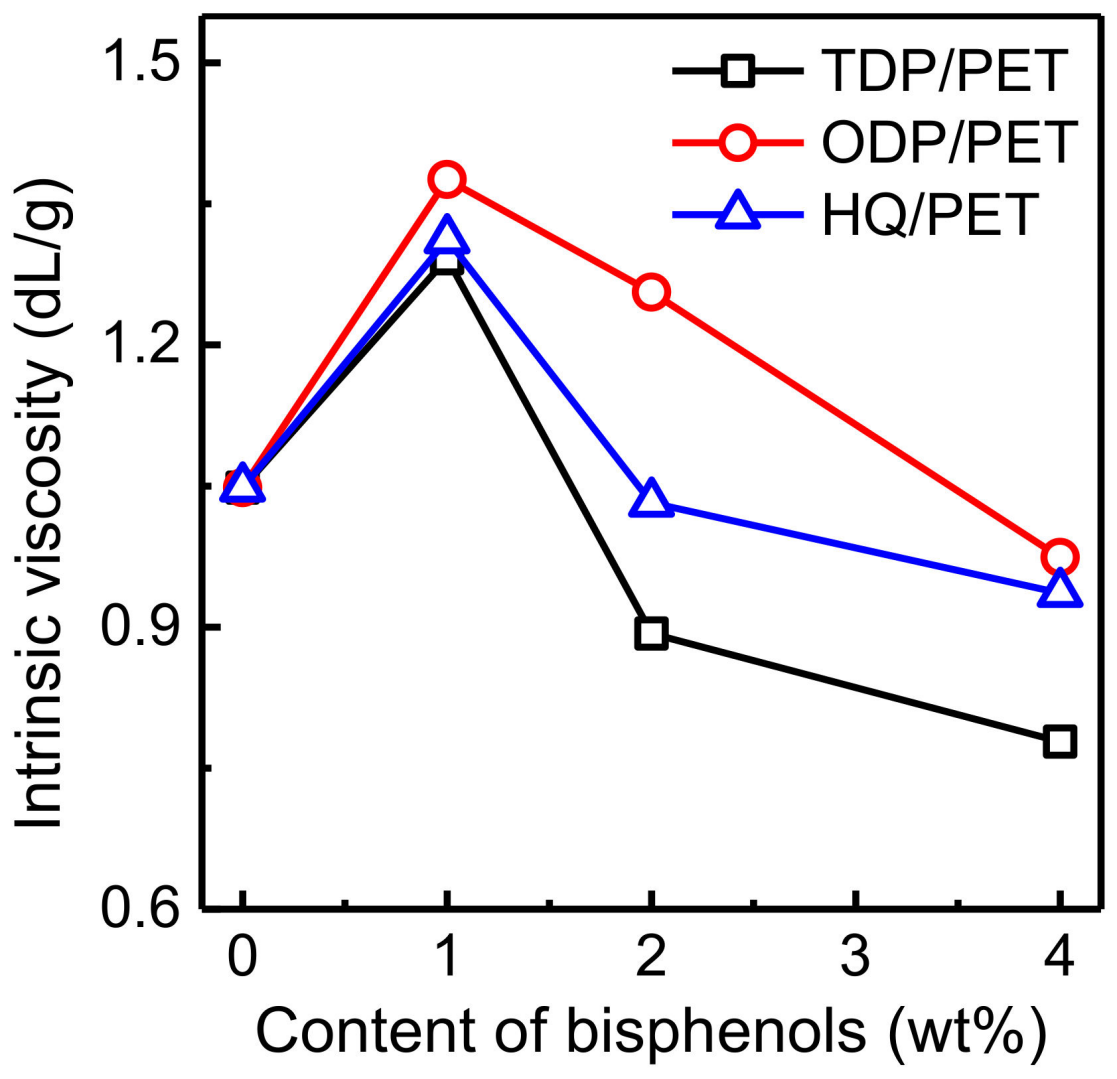
The intrinsic viscosity of PET modified by bisphenol compounds.

The relationship between IV of melt-modified PET and content of bisphenols is shown in Figure 1. To investigate the growth ability of PET molecular chains by melt post-polycondensation in the presence of bisphenol compounds, the bisphenols/PET complexes have been tablet pressing into fixed thickness in advance, which will cause the IV of PET to be slightly lower than the original. It can be seen that the pure PET exhibited an IV of 1.048 dL/g. By reacting different bisphenols with PET, the IV of melt-modified PET first increased to around 1.30 dL/g by addition of 1 wt% bisphenols but decreased with further increase of bisphenols. PET with 4 wt% TDP exhibited the lowest IV of 0.778 dL/g. These results indicate that in the melt post-polycondensation process, the bisphenols were successfully reacted with PET. It is supposed that bisphenols can undergo chain growth with the active end groups of PET and degradation reactions with ester groups on the molecular chain simultaneously. When bisphenols content is low, the chain growth reaction occurred predominantly, which is manifested by a substantial increasement of IV. However, when the content of bisphenols further increased, the degradation reaction held the high ground, which led to a significant decrease in IV. The reaction of bisphenols with PET still needs further confirmation by means of analytical techniques other than IV measurements.

### 3.2. Nonisothermal Crystallization and Melting Behaviors

**Figure 2:**
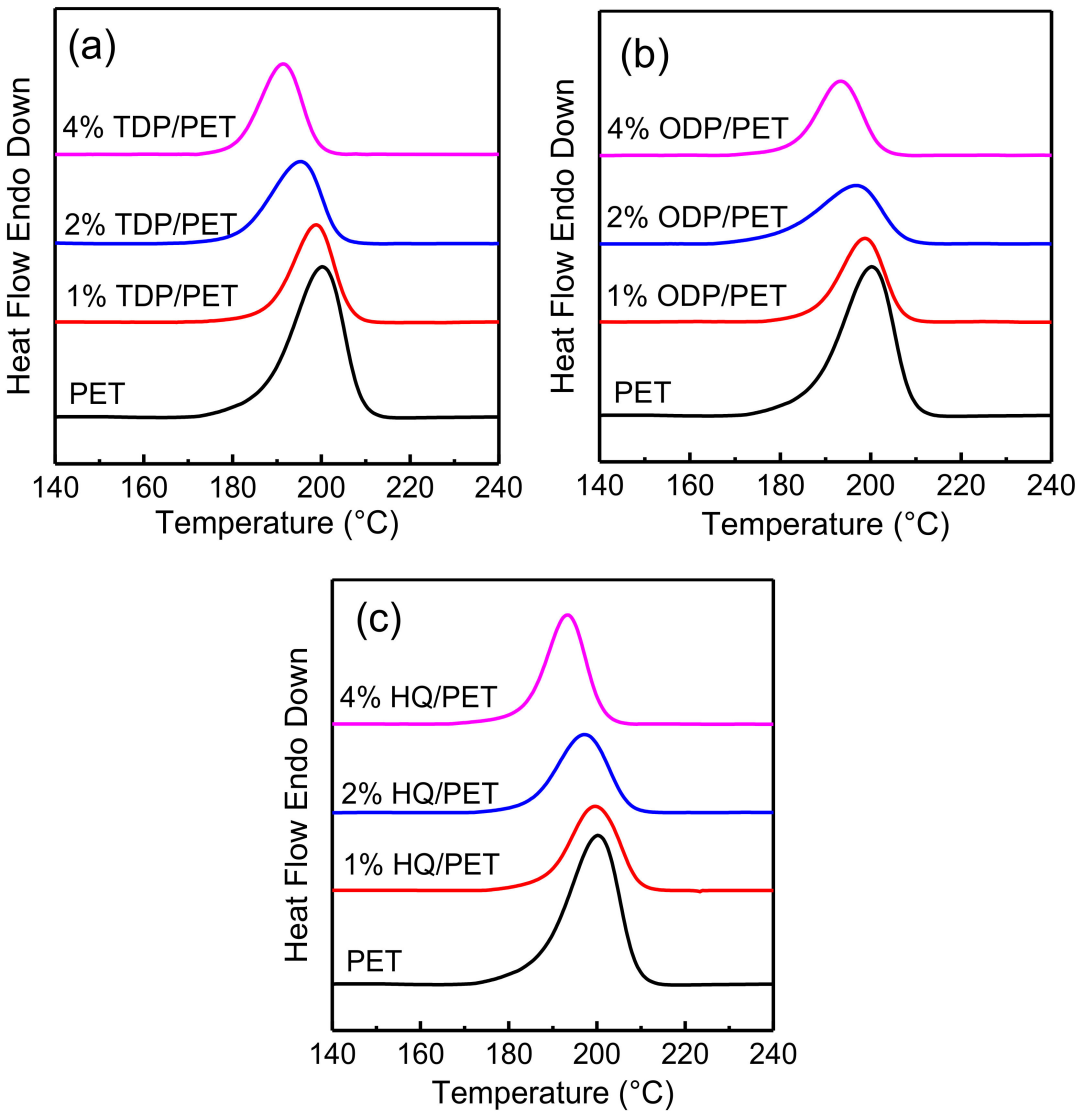
Differential scanning calorimeter (DSC) cooling curves obtained for PET and bisphenols/PET complexes at 10 °C/min: (**a**) TDP/PET, (**b**) ODP/PET and (**c**) HQ/PET.

**Figure 3:**
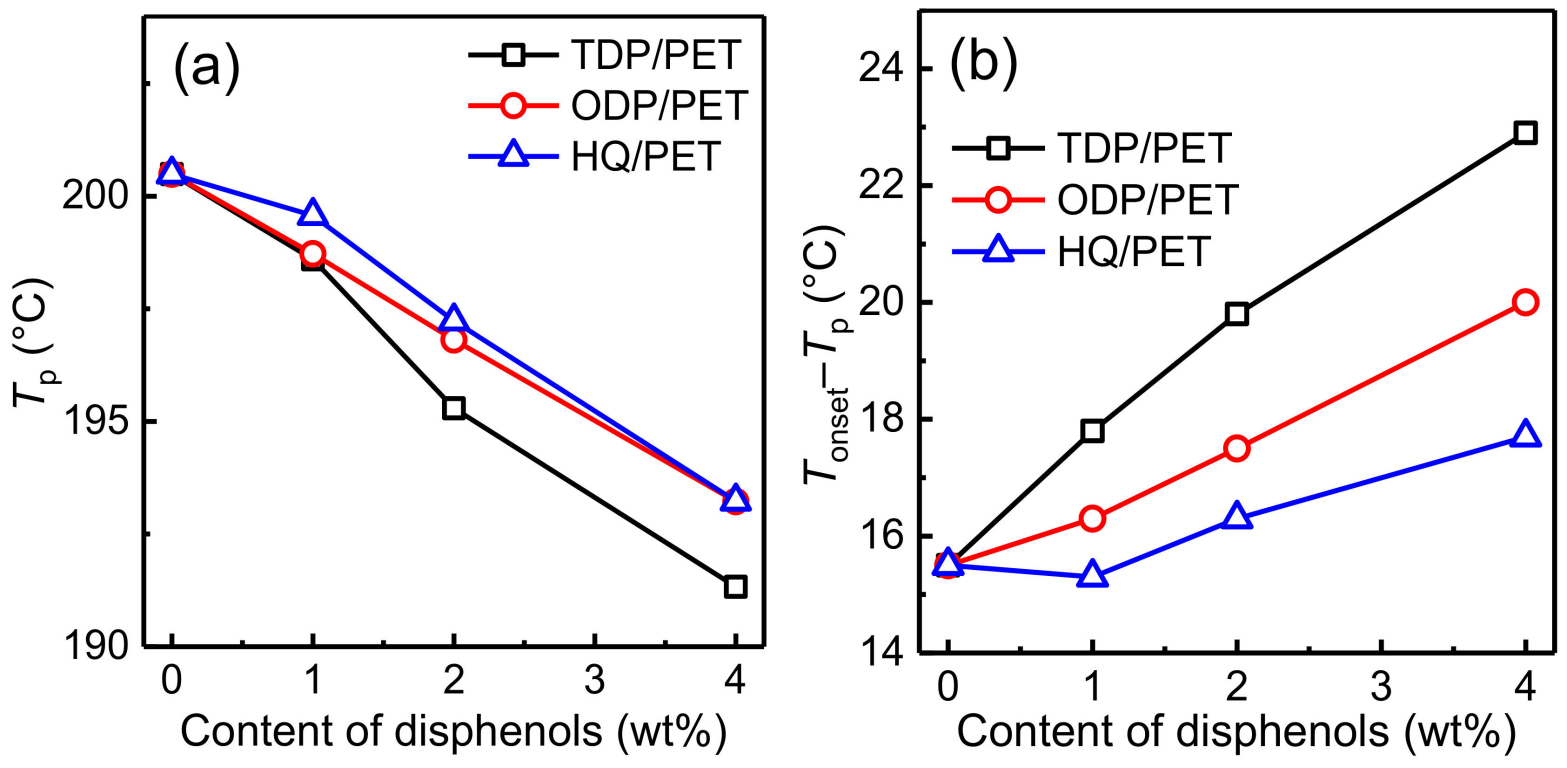
Plot of (**a**) *T*_p_ and (**b**) *T*_onset_−*T*_p_ as a function of content of bisphenols for PET and bisphenols/PET complexes.

The processing forming of PET is usually carried out under non-isothermal conditions. Nonisothermal crystallization kinetics of all samples are investigated via DSC. Figure 2 displays DSC cooling curves of different complexes at the same cooling rate, and corresponding DSC curves of pure PET are also concluded for a better comparison. On the basis of these DSC results, the top of crystallization peaks (*T*_p_) were evaluated and plotted as a function of the weight ratio of bisphenols (Figure 3). Crystallization kinetics of PET were significantly influenced by the presence of bisphenols. With the introduction of bisphenols, the crystallization peak became much broader and the crystallization peak shifted toward the low temperature direction compared to those of pure PET. The higher the bisphenols content is the greater the crystallization peak shifts. Besides, the value ofmelting onset temperature (*T*_onset_)−*T*_p_ can characterize the overall crystallization rate [9]. After addition of bisphenols, *T*_onset_−*T*_p_ value increased, indicative of a lower crystallization rate. Based on *T*_p_ and *T*_onset_−*T*_p_ results, we could draw the conclusion that the introduction of bisphenols had a significant inhibitory effect on crystallization kinetics of PET. It is noticeable that low dosage bisphenols were used in modified PET, and considering the low reactivity between bisphenols and carboxyl end groups of PET chains, a combined effect of bisphenols referring to chain extension, transesterification and intermolecular hydrogen bonds are preferable to understand the results of a feeble inhibitory effect of PET crystallization.

Possible mechanisms of the depressed crystallization rate of PET by the presence of bisphenols were proposed. On one hand, as verified by FTIR measurements, bisphenol molecules are inclined to link to PET chains via hydrogen bonding and may serve as side groups or cross-linking agents. As a result, crystal growth of the polymers will be hindered due to the decreased the chain mobility by the formation of intermolecular hydrogen-bonding between the phenol group and carbonyl. Similar phenomena have been found in PBSA, PBT, PCL, PLLA, PHBV and so on [35,37,38,39,40]. For example, the *T*_p_ of PBSA was around 58.7 °C, and it shifted to 31.8 °C when incorporated 30 wt% of TDP into PBSA, demonstrating that the crystallization rate of PBSA was retarded by TDP [40]. By the presence of talc, the crystallization of PBT was inhibited because of the formation of chemical bonding interactions between talc and PBT [35]. On the other hand, another candidate reason may be related to the occurrence of transesterification between bisphenol and PET. Therefore, the density of rigid benzene ring structure on the molecular chain will increase to some degree of extent, resulting in reduced molecular chain flexibility. Therefore, PET chain is more difficult to be aligned in the crystallization process compared with pure PET.

With a careful comparison between the selected three bisphenols with different structures, it can be seen that degrees of the inhibition effect on the crystallization rate were different. The *T*_p_ of pure PET was around 200.5 °C, and it shifted to 191.3, 193.2 and 193.2 °C when incorporated 4 wt% of TDP, ODP and HQ into PEG, respectively. Besides, *T*_onset_−*T*_p_ for pure PET was 15.5 °C, and it changed slightly into 17.8, 16.3 and 15.8 °C with the addition of 1 wt% of TDP, ODP and HQ, which further increased to 22.9, 20.0 and 17.7 °C when the weight ratio of corresponding bisphenols reached 4 wt%. The highly coincident tendency towards *T*_p_ and *T*_onset_−*T*_p_ values suggest that the effect of TDP was much remarkable that that of ODP and HQ.

From the structural formula of the bisphenols compound in Scheme 1, it is noted that there was only one benzene ring in the HQ molecular structure and it was less than that of TDP and ODP. It is reasonable to expect that when the same amount of bisphenol is involved in the reaction, the increased rigidity of the PET molecular chain introduced by HQ is the least. Moreover, HQ is a non-polar compound, which exerts the least degree of inhibition on the crystallization process of PET. Although both ODP and TDP contain two benzene rings, the electron cloud distribution of the benzene ring in ODP is more uniform. In other words, the molecular chain polarity of TDP is relatively larger, resulting in the most obvious confinement effect on crystallization of PET. Additionally, the main difference between HQ and the other two compounds consists in the molecular size and shape, which affect the rotational motions along the PET-modified chains. Indeed, the two bent bisphenol units of ODP and TDP limit the conformational degree of freedom of polyester chains much more than HQ, causing a slowdown of crystallization and higher decreases of the crystalline level with respect to the HQ units.

**Figure 4:**
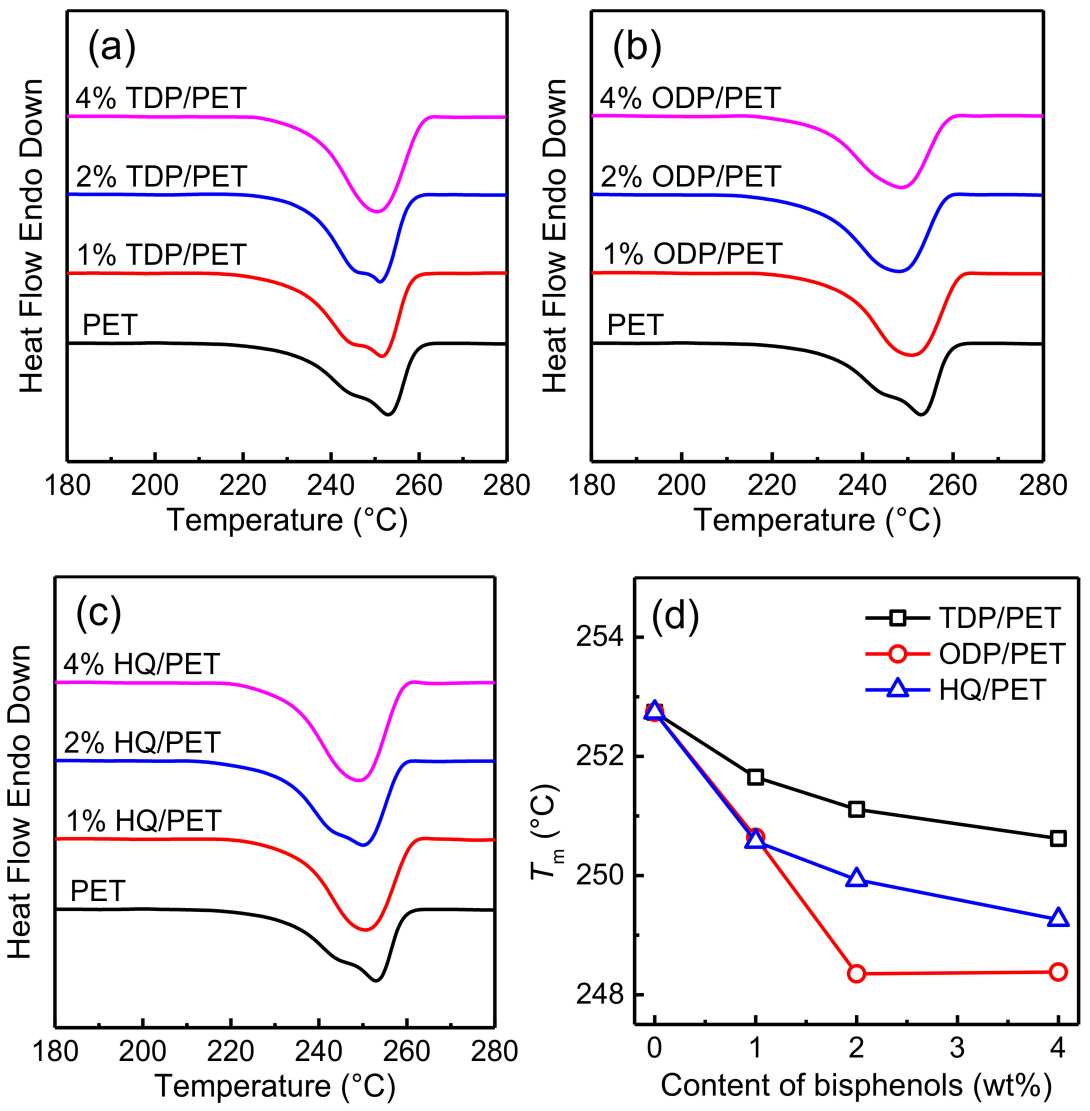
DSC heating curves obtained for PET and bisphenols/PET complexes at 10 °C/min: (**a**) TDP/PET, (**b**) ODP/PET and (**c**) HQ/PET. (**d**) Plots of *T*_m_ as a function of the content of bisphenols.

Figure 4 shows the effect of bisphenols on the melting behaviors of PET. Corresponding melting points are plotted as a function of content of bisphenols. The pure PET exhibited the highest top of melting peak (*T*_m_) value among all samples. As the content of bisphenols increased, the *T*_m_ shifted to lower values. For example, *T*_m_ values for pure PET and TDP modified-PET with 1, 2 and 4 wt% are 252.7, and 251.7, 251.1 and 250.6 °C, respectively. When the amount was the same, the modified PET containing TDP exhibited the higher *T*_m_ value than those of ODP and HQ. This behavior suggests that the incorporation of bisphenols and hydrogen-bonding interaction suppressed the crystallization ability of PET and inhibited the formation of more perfect crystal structure with larger thickness.

### 3.3. Effects of Cooling Rates

**Figure 5:**
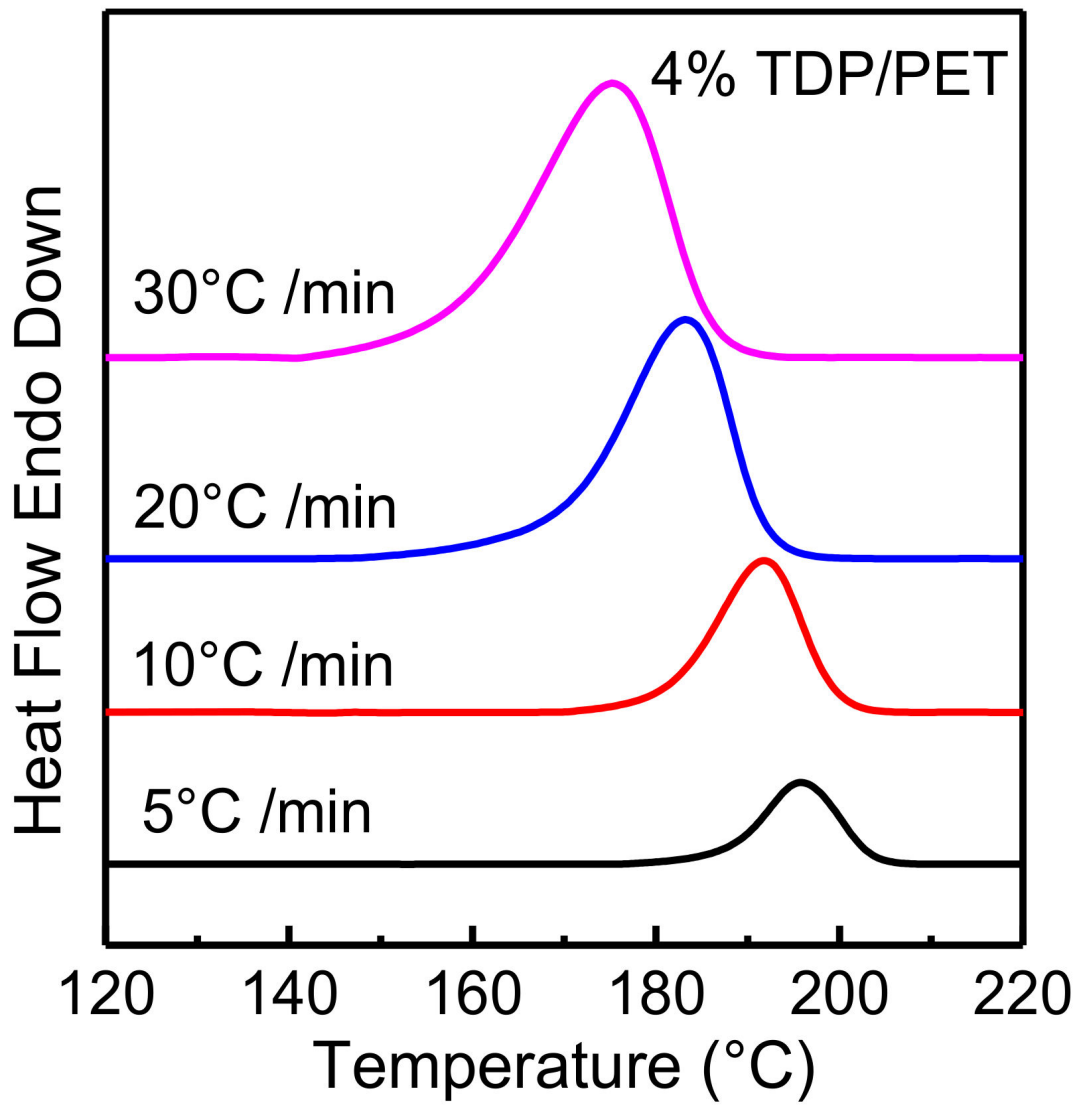
DSC cooling curves at various cooling rates for TDP/PET complexes.

In general, the crystallization behaviors of semicrystalline polymers are functions of both processing and material parameters. Except for the bisphenols type and contents, cooling rate during the nonisothermal crystallization process plays an important role. Figure 5 presents the DSC cooling curve of melt-modified PET with 4 wt% TDPs at different cooling rates. When the cooling rate increased from 5 to 30 °C/min, the peak became broader and the *T*_p_ was shifted to lower values. It is clear that faster cooling rates provided less time for the molecular chain to regularly align and diffuse into the crystal lattice. Therefore, to complete the crystallization process, *T*_p_ was decreased and the crystallization temperature range was broadened. It is well-known that crystallization is an exothermal process, it is needed to remove heat from the growth front in order to allow advancement of crystallization and increases with decreasing temperature [41]. Therefore, the higher the cooling rate during crystallization, that is the lower the external temperature, the higher the crystallization rate and the crystalline level.

In order to obtain important parameters extracted from DSC thermograms to further shed light on the effects of bisphenols and cooling rates on crystallization kinetics of the modified-PET systems. Non-isothermal crystallization data with various cooling rates (5–30 °C/min) of pure PET and its complexes are dealt with the Avrami model according to the following equations [42]:

(1) $$1-X_{t}=\exp(-Z_{t}t^{n})$$

(2) $$\mathrm{lg}[-\ln(1-X_{t})]=\mathrm{lg}Z_{t}+n\mathrm{lg}t$$

where *X_t_* is the crystallinity at time t, *Z_t_* is the rate parameter and *n* is the Avrami exponent obtained from the slope and intercept of the plot of lg[−ln(1 − *X_t_*)] versus lgt. *Z_t_* determines the crystallization rate and *n* indicates the mechanism of nucleation and crystal growth dimensions.

The modified form of the rate parameter characterizing the kinetics of the nonisothermal crystallization process by introducing a cooling rate (*Φ*) into the Avrami equation is given as follows [43]:

(3) $$\mathrm{lg}Z_{c}=\frac{\mathrm{lg}Z_{t}}{\phi }$$

Finally, the main kinetic parameters of half crystallization time (*t*_1/2_) can be obtained by Equation (4):

(4) $$t_{1/2}={\left(\ln2/Z_{c}\right)}^{1/n}$$

**Figure 6:**
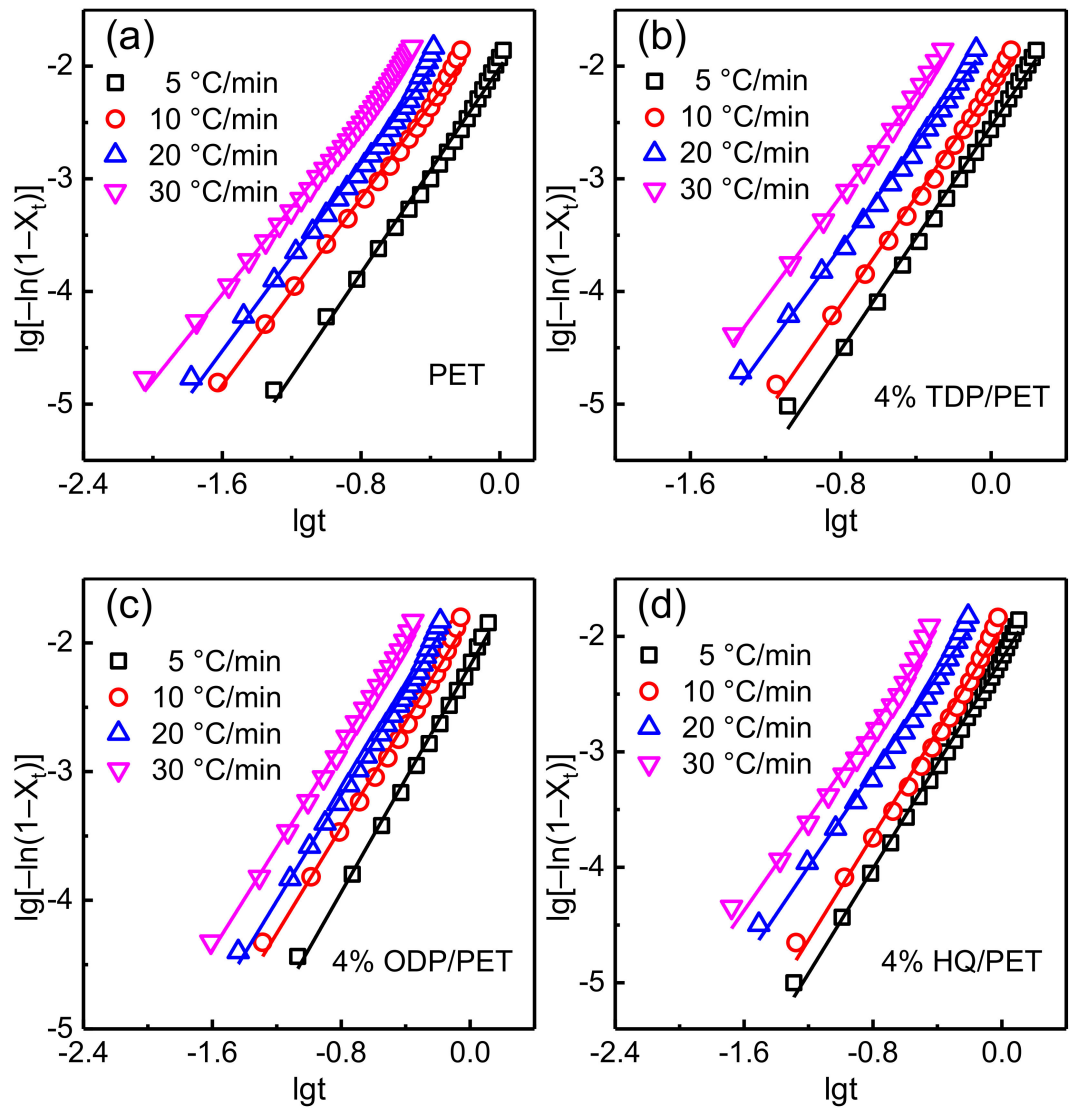
Plots of lg[−ln(1 − *X_t_*)] against lgt for (**a**) PET, (**b**) TDP/PET, (**c**) ODP/PET and (**d**) HQ/PET at various cooling rates with a weight fraction of 4 wt%.

The plots of lg[−ln(1 − *X_t_*)] against lgt and corresponding fitting line for pure PET and modified-PET systems with 4 wt% bisphenols are shown in Figure 6. It is seen that a good linear relationship was obtained in the early stages of crystallization for all samples under various cooling rates, even with incorporation of 4 wt% TDP, ODP and HQ in PET matrixes. This result indicates that the presence of TDP, ODP and HQ exhibited a negligible effect on the induction periods. However, there are upward deviations in the later stages of crystallization at longer times, which is reported in previous literature [13]. The deviation is mainly attributed to the impingement and the nonlinear growth mode of the secondary crystallization in the later crystallization process. The curve shifted as the alternation of the cooling rate because the larger cooling rate would increase the degree of subcooling, thus accelerated the mobility of molecular chains.

**Figure 7:**
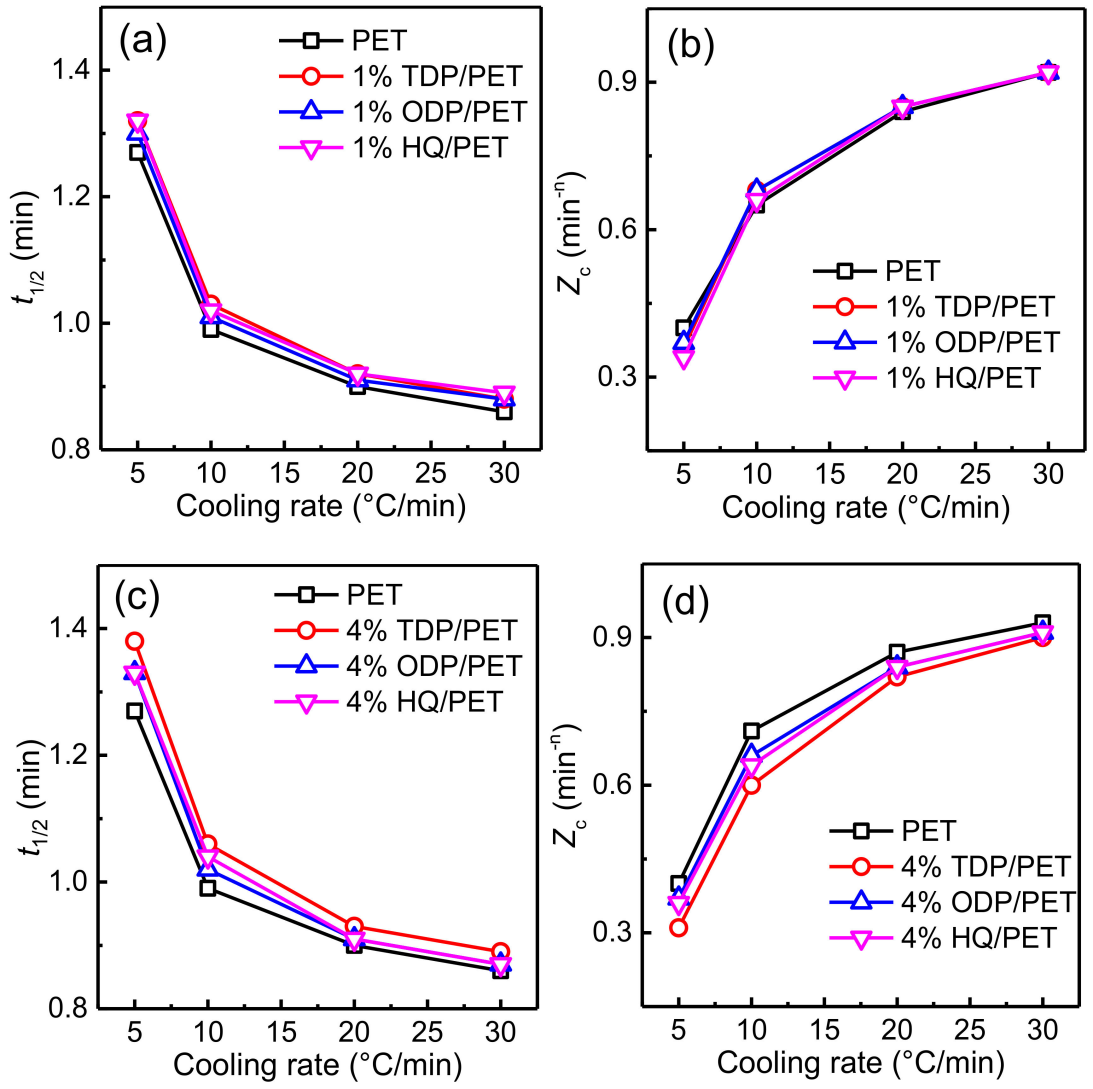
Plots of (**a**,**c**) *t*_1/2_ and (**b**,**d**) *Z*_c_ as a function of the cooling rate for PET and bisphenols/PET complexes.

The values of *t*_1/2_ and *Z*_c_ parameters for pure PET and modified-PET systems with 1 and 4 wt% bisphenols are plotted as a function of cooling rate (Figure 7). With the increase of the cooling rate, the values of *Z*_c_ increase and *t*_1/2_ decrease significantly, which indicates that the crystallization rate of PET increased with the increase of the cooling rate. For pure PET, as the cooling rate rose from 5 to 30 °C/min, the values of *Z*_c_ and *t*_1/2_ were changed from 0.40 min^−n^ and 1.27 min to 0.93 min^−n^ and 0.86 min, respectively, while those for and TDP modified-PET with a TDP content of 4 wt% were altered from 0.31 min^−n^ and 1.38 min to 0.9 min^−n^ and 0.89 min, respectively. Therefore, based on the results of *Z*_c_ and *t*_1/2_, we could draw conclusion that the presence of bisphenols did not change dependence of crystallization kinetics on cooling rates.

In comparison with pure PET under the same cooling rate, by addition of 1 wt% bisphenols, the crystallization rate did not change significantly as the *Z*_c_ and *t*_1/2_ of PET/bisphenols complexes were comparable with those of pure PET, while lower values of *Z*_c_ and higher values of *t*_1/2_ were obtained for all systems with 2 wt% bisphenols. By incorporation of 4 wt% of bisphenols to PET, the crystallization rate was depressed further due to decreased chain mobility by the higher interactions of hydrogen bonding and larger content of benzene rings in molecular chains. Besides, compared to ODP and HQ-modified PETs, *t*_1/2_ and *Z*_c_ of the same amount of TDP-modified PET exhibited the largest differentials under various cooling rates due to the more significant effects on chain mobility, which was highly consistent with the data of *T*_p_ and *T*_onset_−*T*_p_. For instance, when the cooling rates were 5 °C/min, *t*_1/2_ and *Z*_c_ of 4 wt% ODP and HQ-modified PETs were 8.2 and 7.9 min and 0.37 and 0.36 min, respectively, while they increased and decreased to 9.3 and 0.31 min in the case of TDP-modified PET. From the data in the Table 1, *n* value of pure PET ranged from 1.93 to 2.28, and that of modified PET with various contents range from 1.99 to 2.58 upon measured cooling rates. Generally, *n* is depending on both the nature of nucleation and growth geometry of crystals [44]. This indicates that the introduction of bisphenol had almost no significant effect on the nucleation and growth geometry of PET. Besides, it is observed that the variation of *n* parameter for TDP, OPD and HQ from pure PET was similar and had no distinctive dependence on the content of bisphenols.

### 3.4. Crystalline Structure and Crystallinity

**Figure 8:**
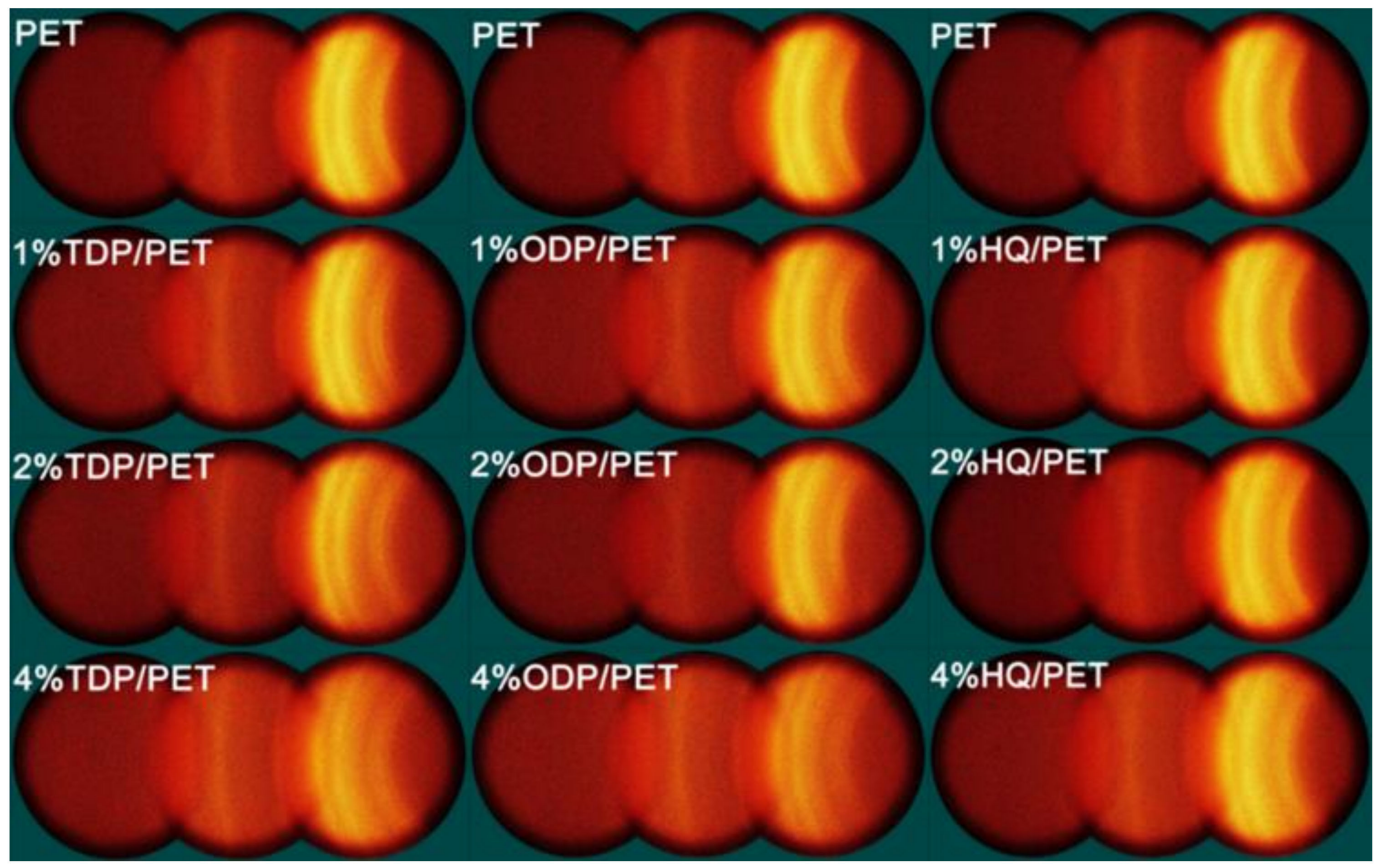
Selected 2D wide-angle X-ray diffraction (WAXD) patterns of PET and bisphenols/PET complexes.

2D WAXD patterns of pure PET and its bisphenols complexes after crystallization and the crystallinity are shown in Figure 8. The image was composed of a superposition of three circular diffraction patterns with a 2*θ* from 2.95 to 76°. There were three diffraction rings of PET in the pattern, where the three distinct bright rings were corresponding to (010), (−110) and (100) crystallographic plane of PET [45]. It can be seen that the brightness of diffraction ring became gradually darker as the content of bisphenols increased, suggesting a reduction of crystallinity. Besides, the variation in brightness of the diffraction ring become more distinctive for TDP than those for ODP and HQ when compared to pure PET, which may be related with their molecular structures.

**Figure 9:**
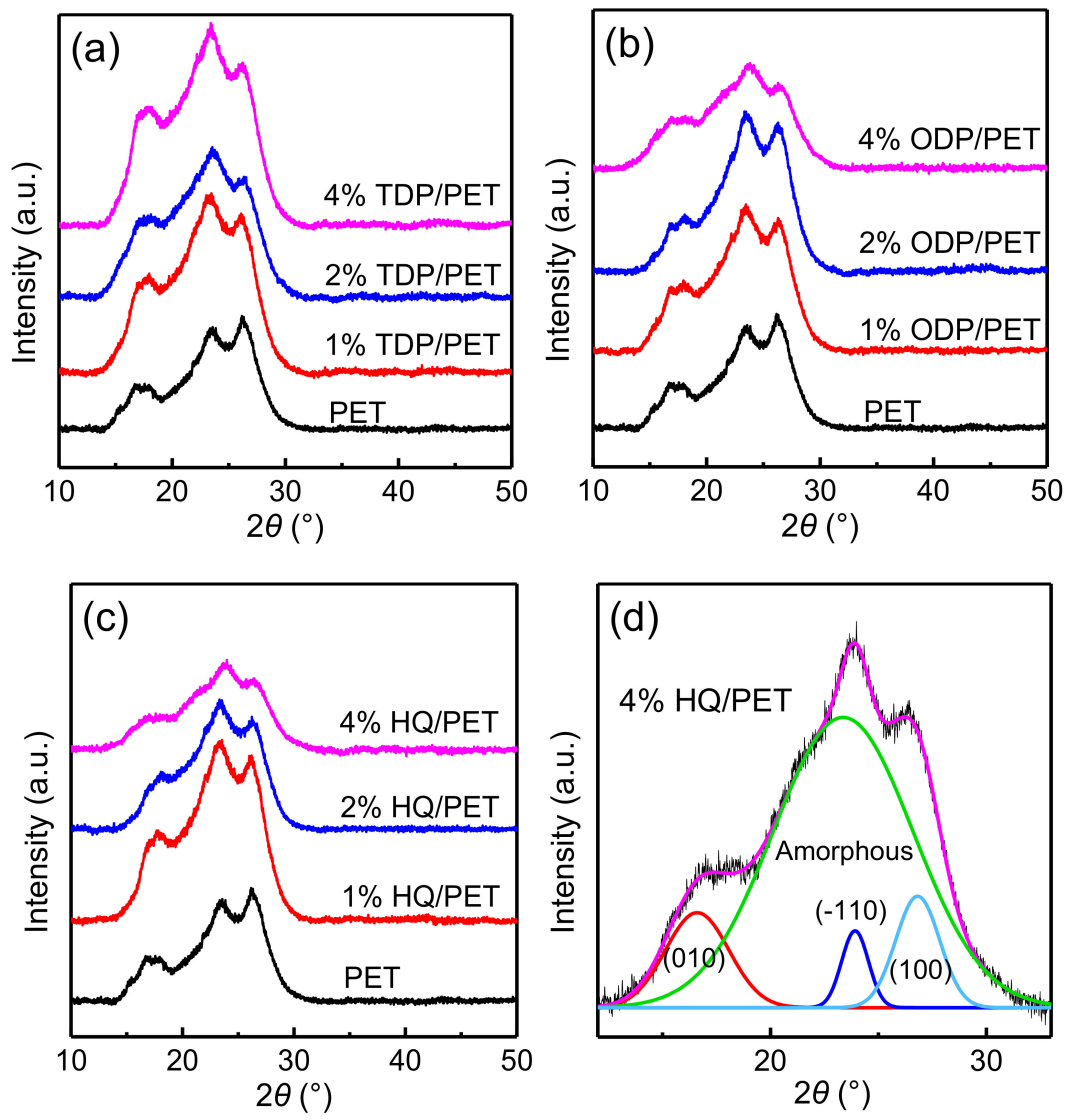
WAXD patterns for (**a**) TDP/PET, (**b**) ODP/PET, (**c**) HQ/PET and (**d**) typical fitting process with Pseudo-Voigt function.

**Figure 10:**
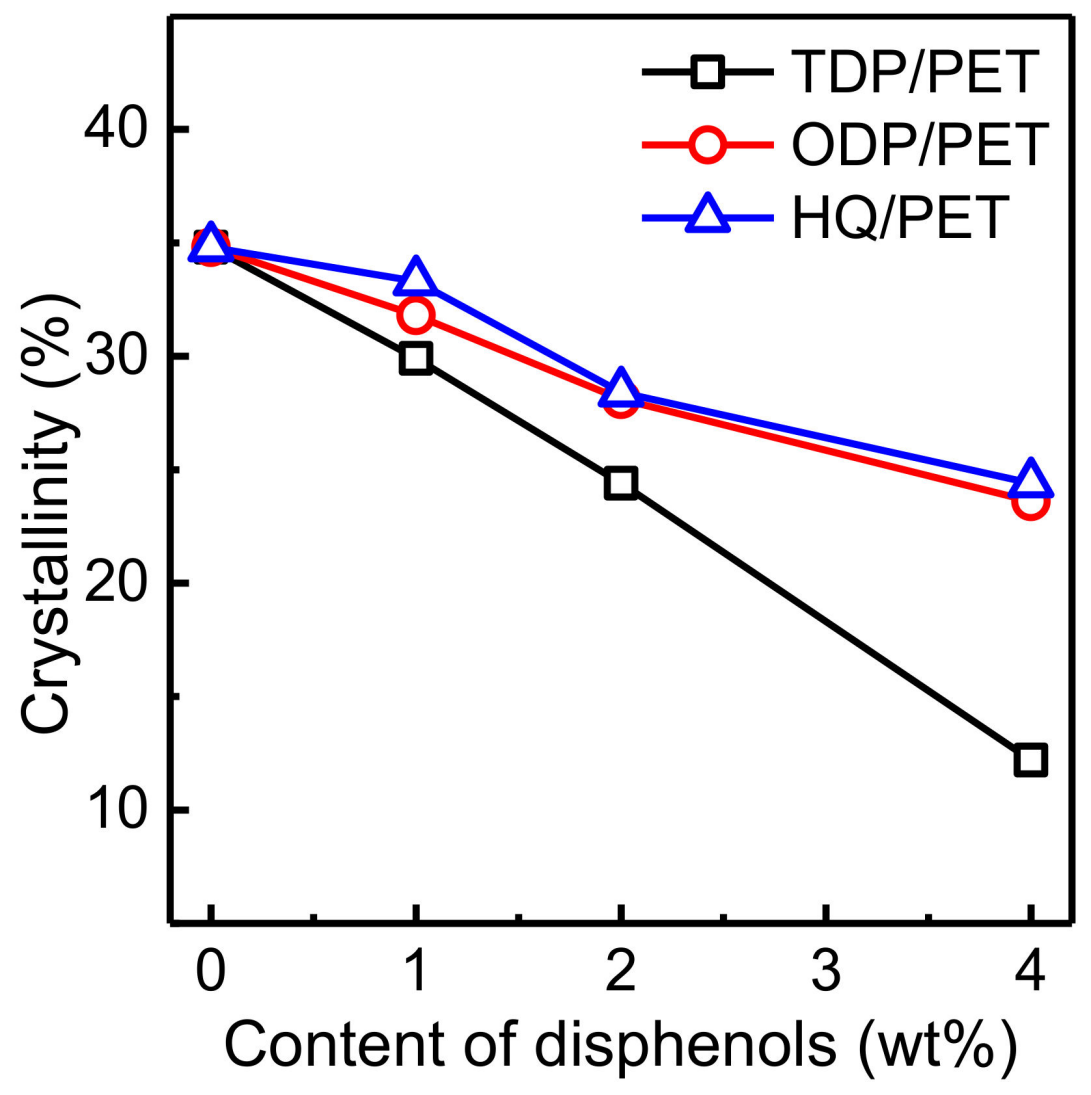
Composition dependence of *X*_c_ of PET and bisphenols/PET complexes.

Next, the 2D XRD patterns were converted into 1D profiles to obtain a peak fitting diagram. The crystallinity was calculated from the peak area and was plotted as a function of bisphenols content. After modification of PET, the main diffraction peaks were located at almost the same 2*θ* as pure PET, and no new diffraction peak is observed, which suggests that the inclusion of bisphenols did not alter the crystalline structure of PET. The sharpness and the intensity of diffraction peaks of (010), (−110) and (100) crystallographic planes decreased gradually with increasing bisphenol content (Figure 9). At a content of 1 and 2 wt%, the PET shows low *X*_c_ by adding TDP, but ODP and HQ did not reduce the *X*_c_ as significant as TDP. Moreover, the *X*_c_ difference between TDP and ODP, HQ became much more significant as the contents increased. As shown in Figure 10, for pure PET, *X*_c_ was 34.8%. In contrast, the *X*_c_ decreased to 12.2%, 23.6% and 24.4% by increasing the TDP, ODP and HQ content to 4%. The proper reason was mentioned in previous nonisothermal crystallization analysis that TDP contained two benzene rings in one molecule and its polarity was relatively larger. Therefore, the inhibition of crystallization ability was more obvious due to the highest rigidity of PET molecular chain incorporated with TDP. Compared with the crystallinity measured by DSC, the decrease of crystallinity measured by 2D-WAXD was more obvious, mainly because the two methods use different principles to measure crystallinity. The crystallinity in 2D-WAXD was obtained by measuring the diffraction peak signal of the characteristic crystal surface and peak-differentiating and imitating process. The 2D WAXD patterns of PET could show the whole characteristic crystal surface if it had a relatively perfect crystallization. However, there was no in-depth report on the difference of the crystallinity measurement results of the two. It was still scientifically very appealing.

### 3.5. Thermal Properties

**Figure 11:**
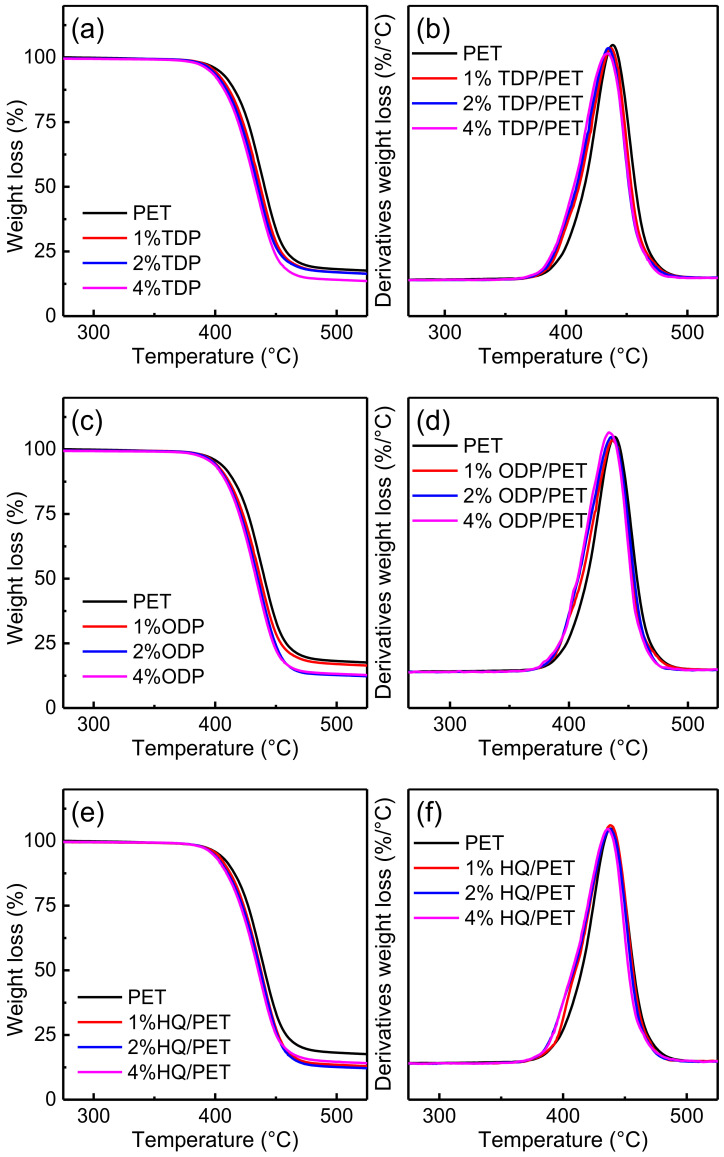
(**a**,**c**,**e**) Weight loss curves and (**b**,**d**,**f**) derivatives weight loss curves for PET and bisphenols/PET complexes.

Figure 11 presents the weight loss and derivative thermogravimetry curves of pure PET and bisphenol-modified PETs. The 5% (*T*_d5%_) mass loss of temperatures and the fastest decomposition rate (*T*_dmax_) are listed in Table 2. It can be seen that the *T*_d5%_ and *T*_dmax_ of PET declined slightly with the addition of bisphenols. As bisphenol content increased, the thermal stability of PET demonstrated a bit decrease, which may be ascribed to the enhanced degradation reaction between the phenolic hydroxyl group and the PET chain. *T*_d5%_ for pure PET was 438.6 °C, and it dropped by the incorporation of TDP, ODP and QH modified-PET with a content of 4 wt%, but the reduction was within 5 °C. These results in Table 2 indicate that the addition of an appropriate amount of bisphenol would not have much effect on the thermal stability of PET.

## 4. Conclusions

In this work, three kinds of bisphenols were successfully introduced into PET by well-dissolved and blended, fixed-thickness compression and melt post-polycondensation. The effects of bisphenols on the melt post-polycondensation process, crystallization kinetic and thermal properties of PET were studied and quantitatively analyzed. With a small amount of bisphenols, the IV of PET was increased, suggesting a promotion on chain growth. However, it tended to decrease when the bisphenol content was further increased due to the dominant degradation reaction. It was found that the crystallization rate and the degree of crystallinity of PET were reduced by inclusion of foreign bisphenols due to the intermolecular hydrogen bonding and the decreased structure regularity. As the content of bisphenols increased, the crystallization rate and crystallinity of PET were further reduced. Besides, compared to ODP and HQ, the inhibition effect of TDP was more obvious, which might be ascribed to the more benzene ring in one molecule and larger polarity. As the cooling rate increased, the values of *Z*_c_ increased and *t*_1/2_ decreased significantly, indicating that the crystallization rate of PET was increased. The petty differences of thermal decomposition temperature between PET and PET/bisphenols complexes demonstrated the introduction of bisphenols had little effect on thermal stability of PET, regardless of the kind of bisphenols.

## Figures and Tables

**Table 1:** Values of n for PET and bisphenols/PET complexes.

Sample	Cooling Rate			
	5 °C/Min	10 °C/Min	20 °C/Min	30 °C/Min
PET	2.28	2.08	2.09	1.93
1% TDP/PET	2.35	2.20	2.26	2.21
2% TDP/PET	2.49	2.39	2.22	2.29
4% TDP/PET	2.48	2.41	2.30	2.27
1% ODP/PET	2.37	2.38	2.22	2.26
2% ODP/PET	2.58	2.52	2.48	2.31
4% ODP/PET	2.20	2.06	2.05	1.99
1% HQ/PET	2.58	2.57	2.32	2.35
2% HQ/PET	2.34	2.38	2.12	2.12
4% HQ/PET	2.27	2.26	2.06	2.00

**Table 2:** Thermal properties of PET and bisphenols/PET complexes.

Bisphenol Content (wt%)	/	TDP			ODP			HQ		
	0	1	2	4	1	2	4	1	2	4
T_dmax_ (°C)	438.6	437.2	435.2	434.2	436.7	436.0	434.0	438.3	438.1	436.7
T_d5%_ (°C)	402.6	399.1	397.1	394.9	399.1	398.4	396.8	401.1	398.4	397.9
